# Lister and the Edinburgh Students

**Published:** 1877-04

**Authors:** 


Lister and the Edinburgh Students.—Our contemporary, the "Scotsman," says the "Medical Times and Gazette," relates, apparantly in all seriousness, an occurrence in the theatre of the Regius Professor of Clinical Surgery, Edinburg, which could only be fittingly described by a poet. A rumor had got abroad that the Regius Professor was about to be tempted away from Edinburgh by the offer of an honorable post of usefulness and distinction in London. The students of the University naturally were alarmed and grieved at the idea of such a loss to themselves and their Alma Mater; and they presented to the Professor an address, signed by 700 of them, in which, among other things, they declared that the loss would be irreparable—a way of complimenting the man at the expense of the University, which may very well be allowed to enthusiastic young students. Then the Professor replied, and in a strain that could not but charm his pupils and admirers. As he had had no actual offer yet, and therefore it was not easy to say much on the subject, but at least he could say this, that "there existed nothing in London at the present time which he considered good enough to call him away from that school." There was no professional chair so potent for good as the chair of clinicl surgery; but according to the experience of his own student days, and the opinion of every distinguished foreigner who visited their school after the schools of London, as compared with the system in Edinburg, was a mere sham—it was simply neglected and slurred over. And then he went on, after some other words, to this grand climax: "If it were certain that I should have the topmost position in London in private practice, and have to teach clinical surgery as it is taught in any London school at the present time, I would certainly not go to London".—*Am. Md. Weekly.*

